# G Protein-Coupled Estrogen Receptor Mediates Cell Proliferation through the cAMP/PKA/CREB Pathway in Murine Bone Marrow Mesenchymal Stem Cells

**DOI:** 10.3390/ijms21186490

**Published:** 2020-09-05

**Authors:** Shu-Chun Chuang, Chung-Hwan Chen, Ya-Shuan Chou, Mei-Ling Ho, Je-Ken Chang

**Affiliations:** 1 Orthopaedic Research Center, Kaohsiung Medical University, Kaohsiung 807, Taiwan; f86225016@ntu.edu.tw (S.-C.C.); hwan@kmu.edu.tw (C.-H.C.); yashuanchou@gmail.com (Y.-S.C.); 2 Regenerative Medicine and Cell Therapy Research Center, Kaohsiung Medical University, Kaohsiung 807, Taiwan; 3 Department of Orthopedics, College of Medicine, Kaohsiung Medical University, Kaohsiung 807, Taiwan; 4 Division of Adult Reconstruction Surgery, Department of Orthopedics, Kaohsiung Medical University Hospital, Kaohsiung Medical University, Kaohsiung 807, Taiwan; 5 Department of Orthopedics, Kaohsiung Municipal Ta-Tung Hospital, Kaohsiung Medical University, Kaohsiung 807, Taiwan; 6 Institute of Medical Science and Technology, National Sun Yat-Sen University, Kaohsiung 807, Taiwan; 7 Department of Medicinal Research, Kaohsiung Medical University Hospital, Kaohsiung Medical University, Kaohsiung 807, Taiwan; 8 Department of Physiology, College of Medicine, Kaohsiung Medical University, Kaohsiung 807, Taiwan; 9 Department of Marine Biotechnology and Resources, National Sun Yat-Sen University, Kaohsiung 807, Taiwan

**Keywords:** GPER-1, BMSCs, estrogen, estrogen receptor

## Abstract

Estrogen is an important hormone to regulate skeletal physiology via estrogen receptors. The traditional estrogen receptors are ascribed to two nuclear estrogen receptors (ERs), ERα and ERβ. Moreover, G protein-coupled estrogen receptor-1 (GPER-1) was reported as a membrane receptor for estrogen in recent years. However, whether GPER-1 regulated osteogenic cell biology on skeletal system is still unclear. GPER-1 is expressed in growth plate abundantly before puberty but decreased abruptly since the very late stage of puberty in humans. It indicates GPER-1 might play an important role in skeletal growth regulation. GPER-1 expression has been confirmed in osteoblasts, osteocytes and chondrocytes, but its expression in mesenchymal stem cells (MSCs) has not been confirmed. In this study, we hypothesized that GPER-1 is expressed in bone MSCs (BMSC) and enhances BMSC proliferation. The cultured tibiae of neonatal rat and murine BMSCs were tested in our study. GPER-1-specific agonist (G-1) and antagonist (G-15), and GPER-1 siRNA (siGPER-1) were used to evaluate the downstream signaling pathway and cell proliferation. Our results revealed BrdU-positive cell counts were higher in cultured tibiae in the G-1 group. The G-1 also enhanced the cell viability and proliferation, whereas G-15 and siGPER-1 reduced these activities. The cAMP and phosphorylation of CREB were enhanced by G-1 but inhibited by G-15. We further demonstrated that GPER-1 mediates BMSC proliferation via the cAMP/PKA/p-CREB pathway and subsequently upregulates cell cycle regulators, cyclin D1/cyclin-dependent kinase (CDK) 6 and cyclin E1/CDK2 complex. The present study is the first to report that GPER-1 mediates BMSC proliferation. This finding indicates that GPER-1 mediated signaling positively regulates BMSC proliferation and may provide novel insights into addressing estrogen-mediated bone development.

## 1. Introduction

Estrogens play important physiological roles in skeletal systems. Bone is a dynamically remodeling organ and estrogen plays an important role in regulating bone remodeling [1,2,3,4,5]. Furthermore, recent reports indicate that estrogen receptor mediation is required for osteogenesis in osteogenic lineage cells [4,6]. Estrogens have traditionally been considered to act mainly through estrogen receptor (ER) α and ERβ that belong to the nuclear steroid hormone receptor superfamily [7,8]. However, recent studies have reported that G protein-coupled ER (GPER-1), also known as G protein-coupled receptor 30, is a membrane receptor for estrogen that promotes estrogen-dependent activation via extranuclear signaling pathways [9,10].

One review report indicated that the effects of bone remodeling are mediated by ERα and ERβ [11]. A report suggested that GPER-1 may also play a role in bone metabolism to alter bone growth [12], but another study showed that using GPER-1 agonist, G1, did not influence tibia and femur growth in ovariectomized C57BL/6 mice [13]. The GPER-1 is expressed in the human growth plate, and the level of expression is high at early stages of puberty but declines during pubertal progression [14]. GPER-1 deficiency was shown to reduce bone growth in female mice [12]. Furthermore, GPER-1 expression has been reported in osteocytes, osteoclasts and osteoblasts [14,15]. These findings suggest that non-genomic estrogen signaling via GPER-1 may exist in bone. On the other hand, estrogens are known to stimulate bone formation, and the effect of estrogen on bone marrow mesenchymal stem cells (BMSCs) differentiation, but not proliferation, is through ERα [16]. BMSCs are known to be important for osteogenesis and bone formation [17]; however, it has not been reported whether GPER-1 plays a role in BMSCs. This study intends to investigate whether GPER-1 mediates BMSC proliferation and its molecular mechanism.

The GPER-1 mediated signal pathways in cancer cells, and normal cells other than osteogenic cells, that have been reported, such as GPER-1 activation, stimulate cyclic AMP (cAMP) production, [9,18] calcium mobilization [19,20], c-Src expression and matrix metalloproteinase (MMPs) activation in breast cancer cells and other tissues [21,22]. These activated MMPs further activate mitogen-activated protein kinase (MAPK) and phosphatidylinositol 3-kinase (PI3K)/Akt pathways [10,21]. GPER-1 was also reported to be activated through estrogen-mediated extracellular signal-regulated kinase 1/2 (ERK1/2) and increase the concentration of cAMP in breast cancers, which further activates protein kinase A (PKA) and phosphorylates cAMP response element-binding protein (CREB) [23,24]. Estrogen promotes the proliferation of the seminoma-like cell line through GPER-1 induction via the cAMP/PKA pathway [25]. In mouse microvascular endothelial cells, the GPER-1 activation was found to reduce endothelial cell proliferation via the c-src/MAPK pathway [26]. Nevertheless, in our recent survey of related studies, the effects and molecular mechanism of GPER-1 on regulating osteogenic cell proliferation have not been reported. In this study, we hypothesized that GPER-1 may regulate the proliferation of BMSCs via the cAMP-related signaling pathway. Therefore, we first examined the cell proliferation in cultured tibia of a neonatal rat by using the GPER-1-specific agonist (G-1) and the GPER-1-specific antagonist (G-15). Furthermore, we examined the effects of GPER-1 on the proliferation of murine BMSCs and investigated the related signaling pathway.

## 2. Results

### 2.1. GPER-1 Promotes Osteogenic Cell Proliferation in Cultured Rat Tibia, as Determined from Ex Vivo Experiments

**Figure 1:**
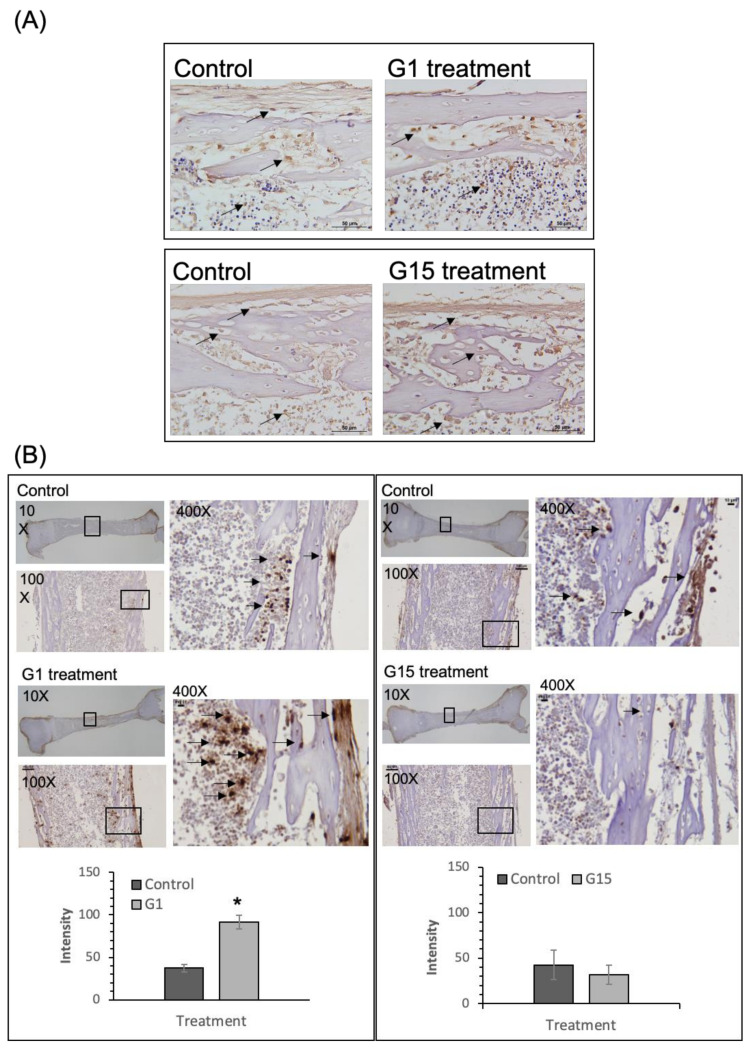
GPER-1 expresses in cultured tibia and mediates the cell proliferation in cultured tibia. (**A**) The GPER-1 positively expressed in cultured tibia in control, G-1 or G-15 treatment group. The brown color (arrows) indicates the GPER-1-positive cells. (**B**) More BrdU-positive cells were shown in the G-1 treatment group than those in control group after 7 days of treatment. It showed a significant difference between control and G-1 treatment group. (* *p* < 0.01 compared of control group). Lesser BrdU-positive cells were shown in G-15 treatment group, but it did not show significant differences between the control and G-15 treatment group (*p* > 0.05). The brown color (arrows) indicates the BrdU-positive cells. (*n* = 6).

The GPER-1 positively expressed in cultured tibia in control, G-1 (500 nM) or G-15 (15 μM) treatment group (Figure 1A). They did not have significant difference between treatments. The GPER-1 positive cells were located at the trabecular, periosteal and endosteal surfaces and in the bone marrow space in cultured tibia. It showed that GPER-1 was positively expressed in osteogenic cells including osteoblasts and BMSCs. We examined whether GPER-1 promotes osteogenic cell proliferation in cultured tibias. The results revealed more BrdU-positive cells in the G-1 group than in the control group (*p* < 0.01 compared of control group) (Figure 1B), but showed no significant differences between the control and G-15 treatment groups (*p* > 0.05). (Figure 1B). These results showed that GPER-1 promotes osteogenic cell proliferation in cultured rat tibia.

### 2.2. Activation of GPER-1 Promotes the Viability and Proliferation of Murine BMSCs

**Figure 2:**
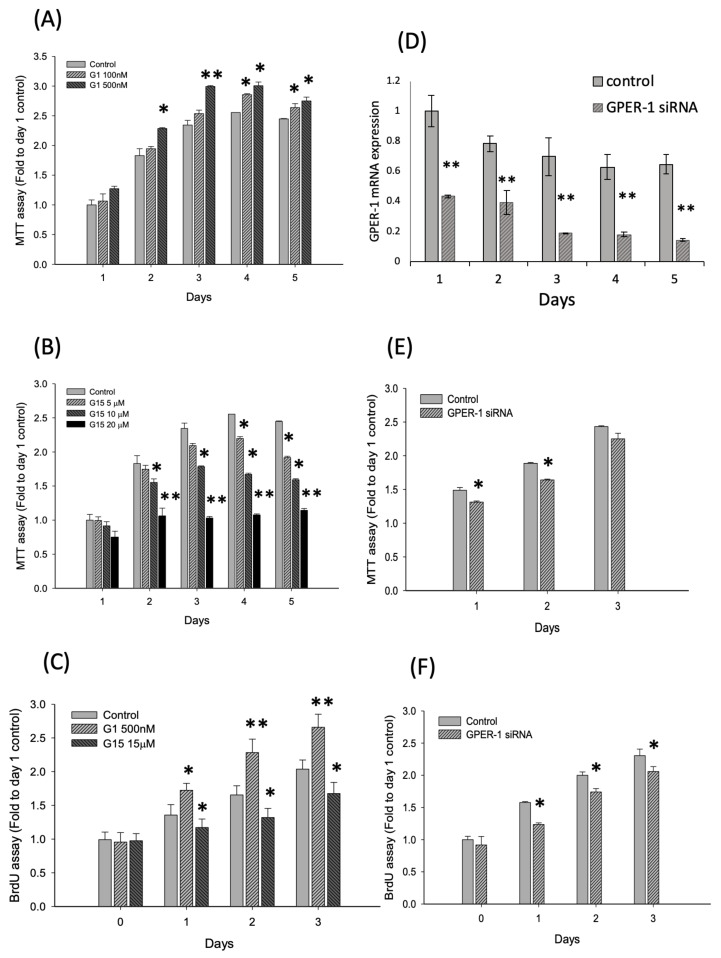
GPER-1 promotes cell viability and proliferation in D1 cells. (**A**,**B**) GPER-1 agonist, G-1, promotes the cell viability and GPER-1 antagonist, G-15, reduces the cell viability in D1 cells by MTT assay. (*p* < 0.05 compare with control group at each day). (**C**) The G-1 promotes cell proliferation and G-15 reduces the cell proliferation in D1 cells by BrdU assay. (**D**) The gene expression of GPER-1 was decreased after GPER-1 siRNA transfection. (**E**) The siRNA GPER-1 reduced the cell viability in D1 cells by MTT assay. (*p* < 0.05). (**F**) The siRNA GPER-1 reduces the cell proliferation in D1 cells by BrdU assay. (*p* < 0.05). (“*” *p* < 0.05; “**” *p* < 0.01; *n* = 4–6).

Before we examined the function of GPER-1 on cell proliferation in murine BMSCs, the GPER-1 protein levels were evaluated. The protein of GPER-1 was expressed through stages of cell proliferation to differentiation (Figure S1). It demonstrated that murine BMSCs express GPER-1 constitutively. For the proliferation experiments, the murine BMSCs (D1 cells, confluence: 20%) were treated with 1 μg/mL nocodazole overnight to synchronize the cell division cycle. Treatment with G-1 (100 and 500 nM) for 1–5 days significantly increased the viability of D1 cells, as determined using the MTT assay (1.17–1.28 folds versus control group at each day, *p* < 0.01; Figure 2A). Moreover, treatment with G-15 (5, 10 and 20 μM) for 1–5 days significantly reduced the viability of D1 cells, as determined using the MTT assay (0.44–0.75 folds versus control group at each day, *p* < 0.01; Figure 2B). Furthermore, BrdU incorporation by D1 cells was significantly increased after G-1 treatment (1.29–1.38 folds versus control group at each day, *p* < 0.01) but was significantly reduced after G-15 treatment 0.79–0.86 fold versus control group at each day, *p* < 0.01; Figure 2C). Similar results were obtained following siGPER-1 treatment. The *Gper-1* gene expressions were reduced to 40%–60% in siGPER-1 group comparing with the control group (Figure 2D). The MTT assay revealed that siGPER-1 treatment reduced the cell viability (0.87–0.92 fold versus control group at each day, *p* < 0.01; Figure 2E). Moreover, siGPER-1 treatment also reduced the cell proliferation, as determined using the BrdU assay (0.78–0.89 fold versus control group at each day, *p* < 0.01; Figure 2F). Overall, these data indicated that GPER-1 activation positively promotes D1 cell viability and proliferation.

### 2.3. Activation of GPER-1 Promotes the Proliferation of Murine BMSCs Through cAMP/PKA/p-CREB Signaling

**Figure 3:**
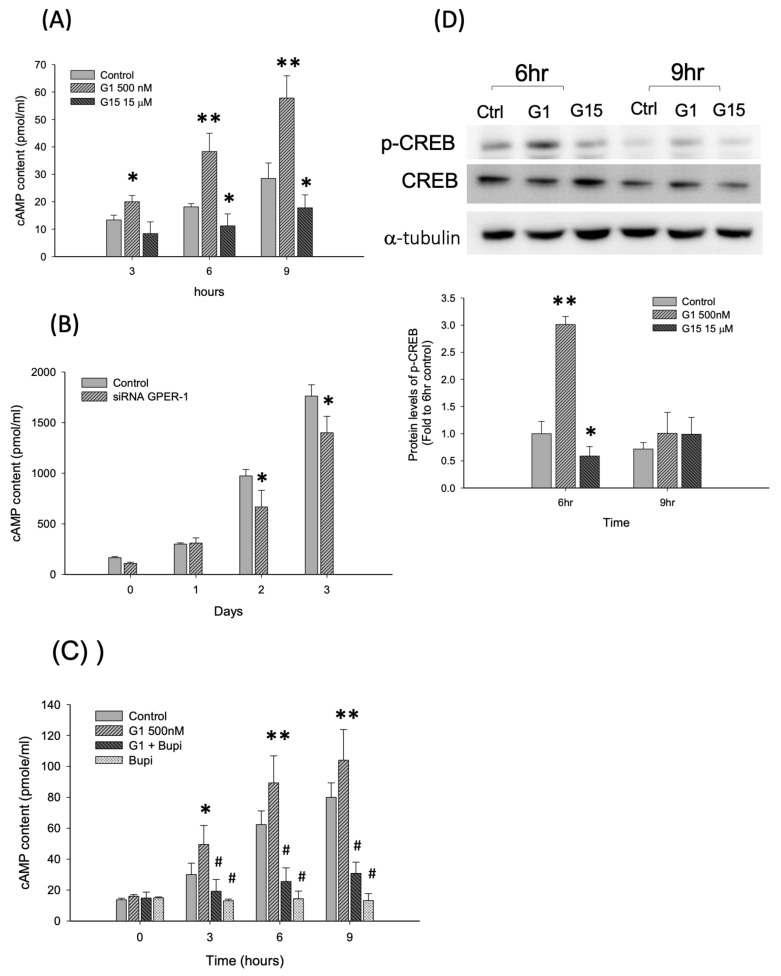
The GPER-1 regulates cAMP/PKA/p-CREB pathway. (**A**) The GPER-1 agonist, G-1, increases the cAMP content and the antagonist, G15, reduces the cAMP content. (**B**) The siRNA GPER-1 reduces the cAMP content in D1 cells. (* *p* < 0.05; ** *p* < 0.01; *n* = 4–6). (**C**) The cAMP inhibitor, Bupi, reduced the G-1 enhanced cAMP content. (* *p* < 0.05 and ** *p* < 0.01 compared with control group; # *p* < 0.05 compared with G1 treatment group.) (**D**) The protein levels of p-CREB was enhanced by G-1 treatment and reduced by G-15 treatment group. (* *p* < 0.05 and ** *p* < 0.01 compared with control group, *n* = 4–6).

The cAMP concentration of D1 cells was determined after G-1 and G-15 treatment. The results revealed that the cAMP concentration increased after G-1 treatment for 3–6 h (1.49–2.11 folds versus control group at each day, *p* < 0.01; Figure 3A) but was reduced after G-15 treatment (approximately 0.62 fold versus control group at each day, *p* < 0.01 Figure 3A). Furthermore, siGPER-1 treatment reduced the cAMP concentration of D1 cells (0.68–0.79 fold versus control group at each day, *p* < 0.01; Figure 3B). The cells were co-treated with a cAMP inhibitor (bupivacaine HCl) and G-1 to evaluate whether the G-1-enhanced cAMP concentration was inhibited by a cAMP inhibitor. The results revealed that G-1 enhanced the cAMP concentration (1.30–1.64 folds versus control group at each day, *p* < 0.01), whereas bupivacaine HCl (100 μM) reduced the G-1-enhanced cAMP concentration (0.29–0.38 fold versus G1 alone, *p* < 0.01; Figure 3C). The protein level of a cAMP downstream signaling molecule, p-CREB, was evaluated through Western blotting. The results revealed that the analyzed expression was increased after G-1 treatment for 6 h (3.01 folds versus controls, *p* < 0.01) but reduced after G-15 treatment (0.58 fold versus controls, *p* < 0.01; Figure 3D). It is not statistically significant at 9 h.

**Figure 4:**
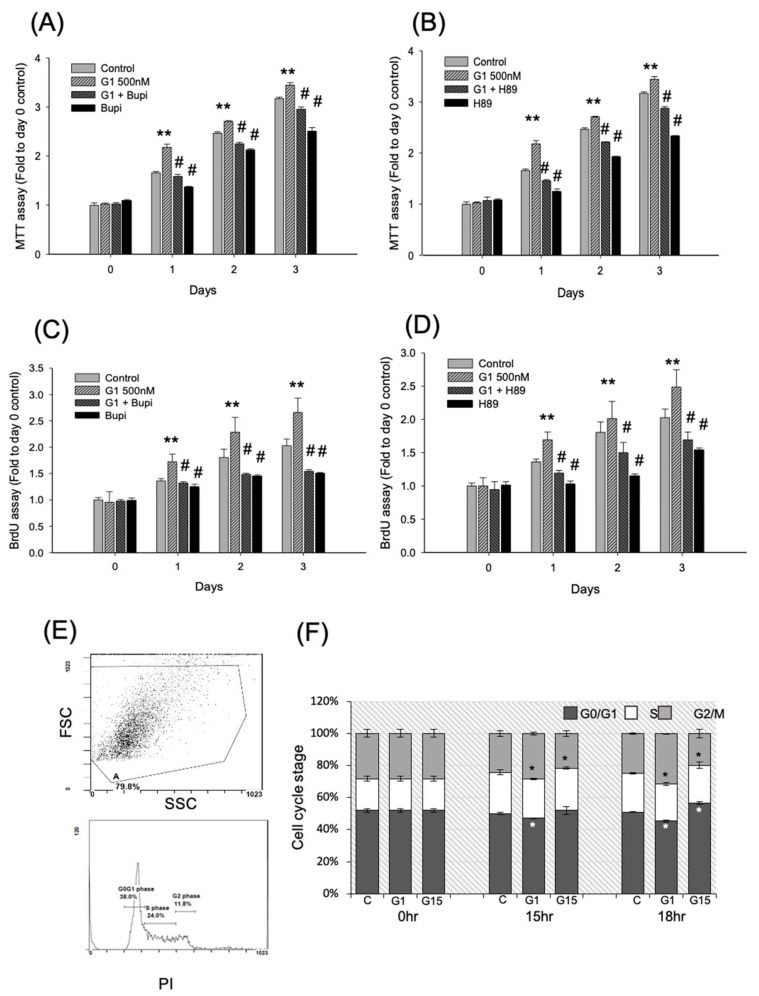
The GPER-1 promoted-cell viability and proliferation was reduced by cAMP inhibitor, Bupi, and PKA inhibitor, H-89. (**A**,**B**) The G-1-promoted cell viability was reduced by Bupi and H89 respectively by MTT assay. (** *p* < 0.01 compared with control group; # *p* < 0.05 compared with G-1 treatment group.) (**C**,**D**) The G-1-promoted cell proliferation was reduced by Bupi and H-89, respectively, by BrdU assay. (** *p* < 0.01 compared with control group; # *p* < 0.05 compared with G-1 treatment group.) (**E**) A representative flow gating strategy of flow cytometry for G-1 at 18hr. (**F**) After treatment of G-1 or G-15, the Cell cycle kinetics was detected by flow-cytometry. The results showed that GPER-1 agonist, G-1, reduced the ratio of G0/G1 phase and promoted cells to G2 phase (* *p* < 0.01 each phase of cell cycle in G-1 group compared with each phase in control group, ANOVA), but the GPER-1 agonist, G-15, reduced the ratio of G2 phase and arrest cell in G0/G1 phase (* *p* < 0.01 each phase of cell cycle in G-15 group compared with each phase in control group,).

The cAMP inhibitor (bupivacane HCl) or PKA inhibitor (H-89) was cotreated with G-1 to evaluate whether GPER-1 activation promotes cell proliferation via the cAMP/PKA/p-CREB pathway; for this, MTT and BrdU assays were used. The results showed that G-1 enhanced the cell viability (approximately 1.30 folds versus controls, *p* < 0.01), but bupivacaine HCl (100 μM) reduced the G-1-enhanced cell viability (0.72–0.85 fold versus G1 alone, *p* < 0.01; Figure 4A). The MTT assay revealed that H-89 (10 μM) also reduced the G-1-enhanced cell viability (0.66–0.83 fold versus G-1 alone, *p* < 0.01 Figure 4B). Moreover, BrdU incorporation by D1 cells showed that bupivacaine HCl and H-89 reduced the G-1-enhanced cell proliferation (bupivacaine HCl: 0.68–0.74 folds versus G-1 alone and H-89: 0.58–0.76 folds versus G-1 alone, *p* < 0.01; Figure 4C,D). These results showed that the activation of GPER-1 promotes the proliferation of D1 cells through cAMP/PKA/p-CREB signaling.

### 2.4. Activation of GPER-1 Promotes Cell Cycle Kinetics

After G-1 or G-15 treatment, the cell cycle kinetics were detected through flow cytometry. A representative flow gating strategy for G-1 at 18hr was showed in Figure 4E. The results revealed that G-1 reduced the ratio of cells in the G0/G1 phase and promoted that of the cells in the G2 phase (*p* < 0.01, ANOVA). However, G-15 reduced the ratio of the cells in the G2 phase and arrested those in the G0/G1 phase (*p* < 0.01; Figure 4F).

### 2.5. Activation of GPER-1 Regulates Cell Cycle Regulators to Promote the Cell Cycle

**Figure 5:**
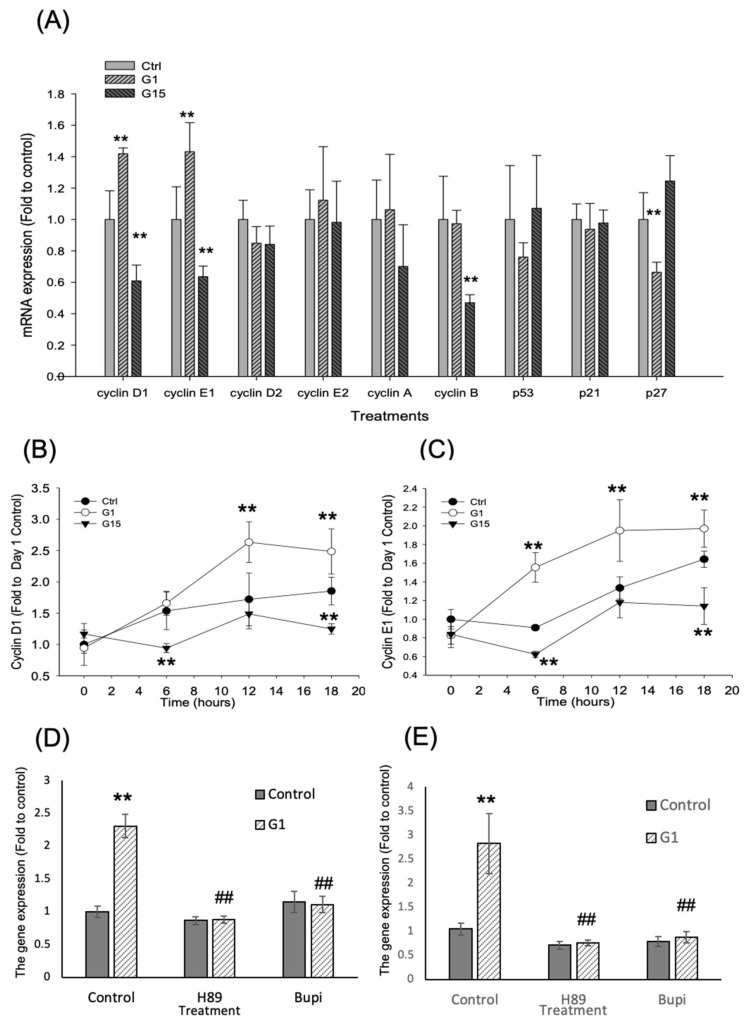
The GPER-1 regulates the gene expression of cell cycle regulators. (**A**) The mRNA expression of cell cycle regulators. (** *p* < 0.01 compared with control group; *n* = 4–6) (**B**) The G-1 promotes the cyclin D1 mRNA expression, but the G-15 reduces the cyclin D1 mRNA expression. (** *p* < 0.01 compared with control group; *n* = 4–6). (**C**) The G-1 promotes the cyclin E1 mRNA expression, but the G-15 reduces the cyclin E1 mRNA expression. (** *p* < 0.01 compared with control group; *n* = 4–6). (**D**) The cyclin D1 gene expression was increased after G1 treatment, but it was reduced by Bupivacaine HCl and H89 (** *p* < 0.01 compared with control group; ^##^ *p* < 0.01 compared with G1 alone group; *n* = 4–6). (**E**) The cyclin E1 gene expression was increased after G1 treatment (**p < 0.01 compared with control group; *n* = 4–6), but it was reduced by Bupivacaine HCl and H89 (##*p* < 0.01 compared with G1 alone group; *n* = 4–6).

Because the activation of GPER-1 promotes the cell proliferation, we examined the expression of cell cycle regulators (Figure 5A). According to the results of mRNA expression, G-1 promoted the mRNA expression of cyclins D1 and E1 (*p* < 0.01, ANOVA), but G-15 reduced that mRNAs of cyclins D1, E1 and B after drug treatment for 12 h (*p* < 0.01, ANOVA). Furthermore, G-1 reduced the CDK inhibitor, p27^kip1^ (*p* < 0.01, ANOVA). However, the gene expression of cyclins D2, E2 and A; p53; and p21 did not vary significantly (Figure 5A).

According to the results, we considered that cyclin D1 and E1 may play important roles in D1 cell proliferation. Quantitative PCR was performed to determine whether increases in the mRNA of cyclins D1 and E1 were time-dependent. The data revealed that G-1 promoted the mRNA expression of cyclin D1 from 12 to 18 h (*p* < 0.01, ANOVA), but G-15 reduced this expression at 6 and 18 h (*p* < 0.01, ANOVA; Figure 5B). Furthermore, G-1 promoted the mRNA expression of cyclin E1 from 6 to 18 h, but G-15 reduced this expression at 6 and 18 h (*p* < 0.01, ANOVA; Figure 5C). Moreover, the G-1-enhanced the gene expressions of cyclins D1 and E1 (approximately 2.5 folds compared with the control group), which were reduced by 12-h treatment with bupivacaine HCl and H89 alone (*p* < 0.01, ANOVA; Figure 5D,E).

**Figure 6:**
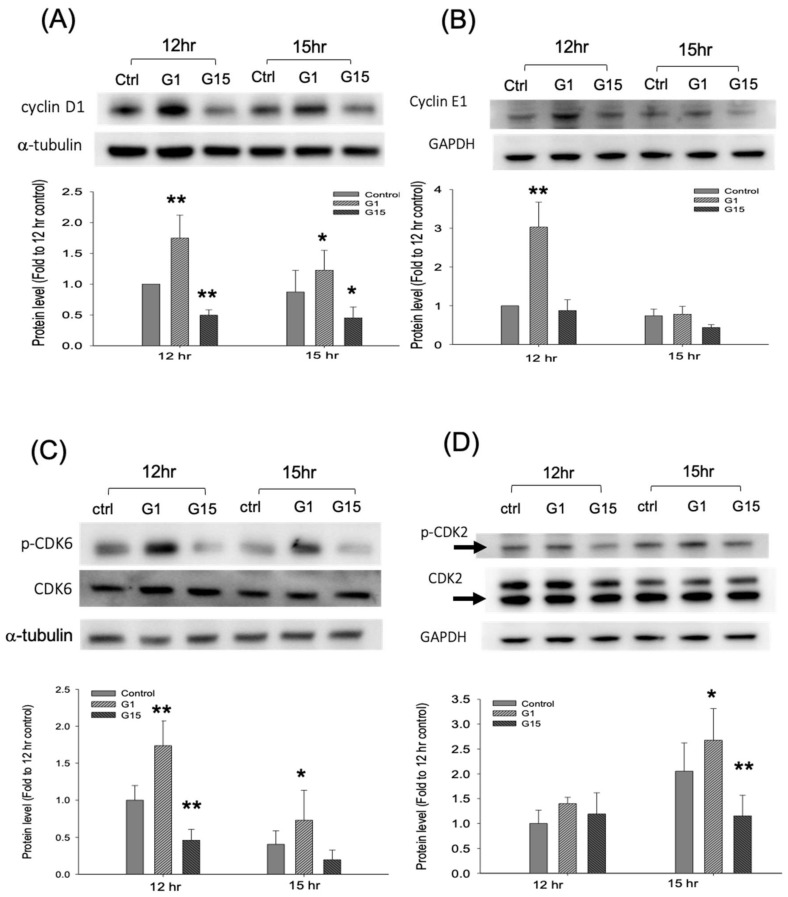
The GPER-1 regulates the protein levels of cyclins and CDKs. (**A**) The G-1 promotes the protein levels of cyclin D1, but the G-15 reduces the cyclin D1 protein levels. (* p<0.05 and ** *p* < 0.01 compared with control group; *n* = 4–6). (**B**) The G-1 promotes the cyclin E1 protein levels, but the G-15 reduces the cyclin E1 protein levels. (** *p* < 0.01 compared with control group; *n* = 4–6) (**C**) The G-1 promotes the p-CDK6 protein levels, but the G-15 reduces p-CDK6 protein levels. The total protein of CDK6 was not significant difference between treatments. (* *p* < 0.05 and ** *p* < 0.01 compared with control group; *n* = 4–6). (**D**) The G-1 promotes the p-CDK2 protein levels, but the G-15 reduces p-CDK2 protein levels. The total protein of CDK2 was not significant difference between treatments. (* *p* < 0.05 and ** *p* < 0.01 compared with control group; *n* = 4–6).

We further examined the protein levels of cyclins and CDKs. The results revealed that the protein levels of cyclins D1 and E1 were enhanced by G-1 but reduced by G-15 at 12 h (*p* < 0.01, ANOVA; (Figure 6A,B). The total CDK2 and CDK6 did not differ significantly between the treatments. However, p-CDK2 and p-CDK6 expression were increased by G-1 treatment (*p* < 0.01, ANOVA) but reduced by G-15 treatment (*p* < 0.01, ANOVA; Figure 6C,D). These data revealed that GPER-1 activation promoted the cell proliferation by enhancing the expression of cyclins D1 and E1, and further enhanced the phosphorylation of CDK2 and CDK6.

## 3. Discussion

**Figure 7:**
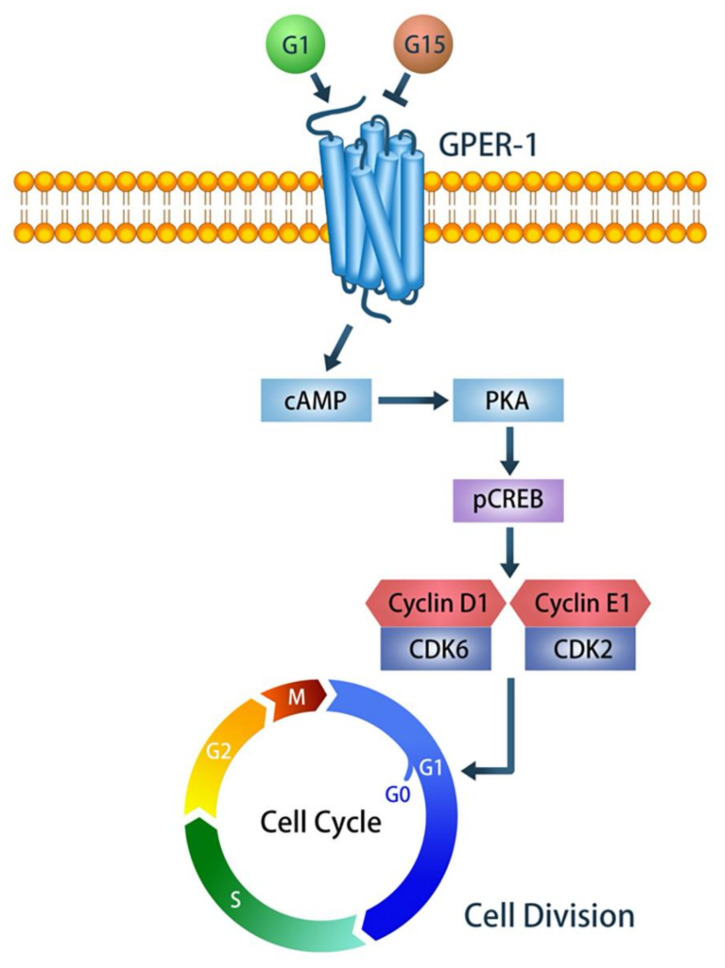
Diagram illustrating the molecular mechanism by which GPER-1 mediation enhances the cAMP/PKA/p-CREB pathway and subsequently up-regulates expression of cell cycle regulators (cyclin D1/CDK6 and cyclin E1/CDK2 complex), and thus promotes BMSC proliferation.

GPER-1 was indicated to express in human osteoblasts, osteocytes, chondrocytes and at growth plates [14]. In this study, we further showed it is also expressed in rat and mice osteogenic cells. GPER-1 has been suggested to play a role in bone development [7,12,27], and mediate the effect of chemicals, such as rutin and ligustilide, on osteogenic differentiation [28,29]. A study showed that GPER-1 activation promotes osteogenesis on the pre-osteoblast cells, MC3T3E1, through the AMPK/anti-acetyl-CoA carboxylase (ACC) pathway [30]. However, to our best knowledge, no study has been reported to investigate the effects of GPER-1 on normal proliferation in osteogenic cells. Therefore, in this study, we investigated whether GPER-1 mediates cell proliferation and studied the related molecular mechanism by using cultured tibia of neonatal rat and murine BMSCs. We demonstrated that GPER-1 is highly expressed in rat neonatal tibia and enhanced proliferation of osteogenic cells at bone surface and in the bone marrow, indicating GPER-1 positively mediates osteogenic proliferation. We further found that GPER-1 mediation enhances the cAMP/PKA/p-CREB pathway and subsequently upregulates the expression of cell cycle regulators (cyclins D1 and E1 and p-CDK2 and p-CDK6), thus promoting the BMSC proliferation (Figure 7). Few studies have investigated whether GPER-1 regulates cell proliferation in mouse-derived neural stem/progenitor or prostate stromal cells [31,32,33] and most related studies have focused on cancer cells [34,35,36]. The present study is the first to report that the GPER-1 mediated signal enhances normal BMSC proliferation.

Osteogenic lineage cells, such as BMSCs and osteoblasts, are important in all phases of bone formation, including cell proliferation and osteogenic differentiation which involves matrix maturation and mineralization [37]. Estrogens are known to regulate the osteogenic differentiation but not cell proliferation through ERα in BMSCs [16]. Our previous study showed simvastatin-stimulated osteogenic differentiation was through ERα but not through GPER-1 [38]. In this study, we found GPER-1 plays an important role in proliferation of BMSCs. Our results revealed that the GPER-1 specific agonist, G-1, promotes the viability and proliferation of murine BMSCs in a dose-dependent manner, whereas the antagonist, G-15, significantly inhibits these activities. These results were evident by BrdU assay for DNA replication to indicate proliferation and MTT assay for mitochondrial function to indicate viability. Among the results, both siGPER-1 and G-15 showed the similar effect on reduction of cell proliferation. However, G-15 treatment showed more severe effect on cell viability inhibition than siGPER-1 treatment did. It may be due to the additional suppressive effect of G-15 on mitochondrial activity other than the antagonism effect on GPER function. GPER-1 activation enhances DNA replication in the osteogenic cells of cultured tibias in our study. The present findings reveal that the GPER-1 activation promotes BMSC proliferation in vitro and in ex vivo.

Since both ERα and GPER-1 are expressed in BMSCs, it is difficult to distinguish the effect via ERα, ERβ or GPER-1 using the nature ligand Estradiol. We used G-1 and G-15 instead of using estradiol to avoid the estrogen effect through receptors other than GPER-1. The serum for the cell cultures in our study is charcoal stripped to remove endogenous estradiol. On the other hand, the presence of phenol red in the media may also act as a weak estrogen-like ligand to confound the results. Theoretically, the most appropriate way is to use charcoal stripped serum with phenol-red-free medium for cell cultures. However, in our previous study [38], charcoal stripped serum combined with phenol-red-free medium as a control medium caused cytotoxity in BMSCs. A previous report also showed the similar phenomena and indicated that E2 was hardly detectable in normal culture medium containing 15% FBS [39]. Therefore, we only used the charcoal stripped serum in this study.

The molecular mechanism of proliferation by GPER-1 mediation in different kinds of cells has been reported by stimulating cAMP production [9,12,18], intracellular calcium mobilization [19,20] and PI3K activation [21,40]. In primary human endometriotic cells, a GPER-1-selective tamoxifen analog, STX, significantly stimulates PI3K and MAPK pathways to promote cell proliferation [41]. Estrogens also promote the proliferation of the seminoma-like cell line through GPER-1 mediation via the cAMP–PKA pathway [25]. However, in mouse microvascular endothelial cells, GPER-1 activation attenuates cell proliferation via the c-src/MAPK pathway [26]. These findings of previous studies showed that GPER-1 regulates the proliferation in kinds of cells via different signaling pathways, such as cAMP/PKA, c-src/EGFR/MAPK and c-src/EGFR/PI3K/Akt pathways [21]. In the present study, in BMSCs, the p-Akt and MAPK protein levels did not differ significantly between the treatment and control groups (data not shown), indicating no involvement of the PI3/Akt or MAPK pathway. We further tested the cAMP-related pathways for GPER-1 promoted proliferation in murine BMSCs because of the importance of cAMP in cell proliferation [42,43]. Our results indicate that the GPER-1 activation increases the concentrations of cAMP and p-CREB. The inhibition of cAMP synthesis or PKA activity reduces the GPER-1-induced cell viability and proliferation. These results demonstrated that GPER-1 mediates the promotion of BMSC proliferation via the cAMP/PKA/p-CREB signaling pathway, but not the PI3K/Akt or MAPK pathway.

Our results further demonstrated that activation of GPER-1 promotes BMSCs cell cycle kinetics by proceeding the G0/G1 to S phase and the S to G2 phase, while suppression of GPER-1 signaling arrests the cell cycle at the G0/G1 phase. The results indicate that the key mechanism underlying the GPER-1-induced cell proliferation is the promotion of the cell cycle at the G0/G1 phase. We further demonstrated that GPER-1 activation promotes the gene expression and protein levels of cyclins D1 and E1, as well as phosphorylation of CDK6 and CDK2. Moreover, the GPER-1-induced gene expression of cyclins D1 and E1 is reduced by the blockade of cAMP synthesis or PKA activity. These results suggest that the GPER-1 promoted functions of cyclins D1 and E1 are associated with CDK6 and CDK2 respectively, and eventually promote cell proliferation. Our result of cyclin D1 mRNA expression elevated starting from 6 h after treatment (non-significant statistic difference) and reaching plateau until 12–18 h. Therefore, the translation event may also occur during 6–12 h, and the increase of cyclin D1 protein can be detected at 12 h time point. Additionally, according to the trend of increases in mRNA and protein of cyclin D1, the effect of G-1 should be on the expression event, but not protein stability.

Cyclins D and E are highly CREB dependent [44], and the CRE for CREB binding is present in the promoter region of cyclin D1 [45]. It indicated cyclin D expression can be regulated directly by CRE-CREP binding on cyclin D promoter. Accordingly, our results suggest that GPER-1 promoted CREB phosphorylation may increase the promoter activity of cyclin D1 in murine BMSCs. It needs more investigation to test whether the effect of GPER-1 on the promoter activity of cyclin D1 or E1 is through promoting CREB phosphorylation. Moreover, it is needed to investigate whether the GPER-1 mediation regulates proliferation and/or differentiation in other osteogenic cells or chondrocytes during bone growth.

In conclusion, our results demonstrated that GPER-1 mediated signaling contributes to BMSC proliferation by upregulating cell cycle regulators, cyclin D1/CDK6 and cyclin E1/CDK2 complexes. GPER-1 activation enhanced the cell cycle proceeding from the G0/G1 to S phase and from the S to G2 phase, thereby promoting cell proliferation. Moreover, our results revealed that the GPER-1-induced cell proliferation depends on cAMP synthesis and PKA activity. It indicated that GPER-1 activation enhances the function of cyclin D1/CDK6 and cyclin E1/CDK2 complexes to promote murine BMSC proliferation via the cAMP/PKA/p-CREB signaling pathway (Figure 7). Our findings suggest that GPER-1 mediated signaling may play an important role in up-regulating BMSC proliferation, that contributed to estrogen regulated skeletal development. It is a novel insight into the molecular mechanisms regarding estrogen-mediated skeletal development.

## 4. Materials and Methods

### 4.1. Cell Proliferation in Ex Vivo Experiments

All animal experiments were approved by The Animal Care and Use Committee of Kaohsiung Medical University (IACUC Approval No. 102194, 26 March 2014). Two male and 4 female 8-week-old Sprague–Dawley rats (250–300 g) were purchased from BioLasco (Taiwan, China) and housed under standard laboratory conditions (24 °C, 12-h light–dark cycle) with ad libitum food and water. The rats were mated to breed neonatal rats. Furthermore, 4-day-old neonatal rats were scarified, and their tibiae were harvested. The tibiae were washed 3 times with antibiotics to avoid contamination; they were then cultured in BGJb medium containing 10% charcoal-striped serum (100 mL of FBS treated with 0.5 g of charcoal and 0.052 g of dextran T-70 for 2 h at 37 °C and then centrifuged at 12,000 rpm for 10 min), 0.5% antibiotics, and 10 μM BrdU. The control group included the left tibia (*n* ≥ 5), and the G-1 (500 nM) or G15 (15 µM) treatment group included the right tibia (*n* ≥ 5). After 7 days, the tibia was harvested and fixed with 10% neutral buffered formalin.

The tibiae were decalcified in 10% formic acid (Sigma-Aldrich, St. Louis, MO, USA) and embedded in paraffin, and 5-μm microsections were prepared from the coronary plane. The cells proliferating in the tibiae were evaluated and stained using a BrdU immunohistochemistry kit (No. ab125306; Abcam, Cambridge, UK). Hematoxylin was using as counterstaining (MHS1–100ML, Sigma-Aldrich, St. Louis, MO, USA) The slides were observed with a Leica-DM1000 microscope (Leica Microsystems, Wetzlar, Germany). The brown color cells were indicated as the BrdU positive cells. For analysis, images were converted to an 8-bit TIF file. The same threshold was set to analyze the DAB intensity by ImageJ software. Each group contained more than six independent samples.

### 4.2. Cell Culture

We use murine BMSCs (D1 cells; CRL-12424™, ATCC) in this study. The cells were cultured in bone medium (low-glucose Dulbecco modified Eagle medium, Gibco BRL and Thermo Fisher Scientific, both Waltham, MA, USA) containing 10% fetal bovine serum (FBS), 50 μg/mL L-ascorbic acid (Sigma-Aldrich, St. Louis, MO, USA), 100 mg/mL nonessential amino acid solution (Thermo Fisher Scientific, Waltham, MA, USA) and 100 U/mL penicillin/streptomycin solution (Thermo Fisher Scientific). The cells were incubated in a humidified atmosphere of 5% CO_2_ at 37 °C [46]. During the drug treatment experiments, serum in the bone medium was replaced with charcoal-striped serum (100 mL of FBS treated with 0.5 g of charcoal and 0.052 g of dextran T-70 for 2 h at 37 °C and then centrifuged at 12,000 rpm for 10 min) to avoid the false estrogen effect. The average doubling time of D1 cells was 16–18 h under the experimental condition [47].

### 4.3. Drug Treatments

GPER-1 specific agonist G-1 (No. 3577; Tocris Bioscience Joins R&D Systems, BristolAvonmouth, UK), GPER-1 specific antagonist G-15 (No. 3678; Tocris Bioscience Joins R&D Systems, BristolAvonmouth, UK), cAMP production inhibitor bupivacaine HCl (Sigma-Aldrich, St. Louis, MO, USA), and PKA-specific inhibitor H89 (No. 2910; Tocris Bioscience Joins R&D Systems, BristolAvonmouth, UK) were dissolved in dimethyl sulfoxide (DMSO) as stock solutions. For the proliferation experiments, sub-confluent cells (20–50%, depending on the experiments) were treated with 1 μg/mL nocodazole overnight to synchronize the cell division cycle. To evaluate the function of GPER-1, D1 cells were treated with G-1 (100 and 500 nM) or G-15 (5, 10 and 20 µM) for 1–5 days. The cells were co-treated with bupivatine HCl (100 µM) or H-89 (10 µM) and G1 to detect whether GPER-1 regulates the cAMP/PKA pathway. All reagents were diluted with charcoal-striped bone medium immediately before the treatments. The final concentration of DMSO in each treatment was less than 0.1%; this concentration was selected to reduce the compound’s influence on the cells. The cells were harvested for the 3-(4,5-dimethylthiazol-2-yl)-2,5-diphenyltetrazolium bromide (MTT) assay on days 1, 3 and 5 and for the BrdU assay on days 1, 2 and 3. The cell cycle kinetics, cAMP concentration assay, and cell cycle regulator gene and protein expressions were evaluated within 18 h.

### 4.4. GPER-1 siRNA Transfection

D1 cells were incubated in antibiotic-free culture medium for 18 h before siRNA transfection. GPER-1 siRNA (siGPER-1; Santa Cruz Biotechnology, Dallas, TX, USA) was transfected to D1 cells by using Lipofectamine RNAiMAX reagent (Invitrogen, Carlsbad, CA, USA); an RNAi-negative universal control was used (Invitrogen, Carlsbad, CA, USA). During transfection, the cells were cultured in non-serum Opti-MEM medium (Life Technologies, Eugene, OR, USA). After transfection for 6–8 h, the culture medium was changed to charcoal-striped bone medium for 1–3 days to evaluate cell viability and proliferation by performing MTT and BrdU assays.

### 4.5. MTT Assay

D1 cells (confluence: 20%, 10^4^ cells/well) were seeded in 24-well plates and cultured to 100% confluence. After drug treatment or siGPER-1 transfection, the cell viability was measured using the MTT assay. In total, 50 μL of MTT solution (5 mg/mL in PBS as the stock solution) was added to each well (the final MTT concentration is 0.5 mg/mL) and then incubated for 4 h at 37 °C. Subsequently, the supernatant was removed, and 100 μL of DMSO was added to each well to dissolve the formazan crystals for 10 min. The 595-nm absorbance of each plate well was read using an ELISA plate reader (Bio-Rad Laboratories Inc., Hercules, CA, USA).

### 4.6. Cell Proliferation Examination (BrdU Assay)

D1 cell proliferation was measured using the Cell Proliferation ELISA BrdU (colorimetric) assay kit (No. 1 647 229, Roche, Penzberg, Germany). Briefly, D1 cells (confluence: 20%, 10^4^ cells/well) were treated with drugs or siGPER-1 in a 24-well plate in a humidified atmosphere at 37 °C. BrdU labeling solution (10 μM) was added to each well, and the cells were further incubated for 6 h at 37 °C. Subsequently, the labeling solution was removed, 200 μL FixDenat was added to each well, and the cells were incubated for 30 min at 15–25 °C. Furthermore, FixDenat solution was thoroughly removed by flicking and tapping; 100 μL of anti-BrdU-POD working solution was added to each well, and the cells were incubated for 90 min at 15–25 °C. The antibody conjugate was removed by flicking, and the wells were rinsed 3 times with washing solution (200–300 μL/well). Substrate solution was added (100 μL/well), and the cells were incubated at 15–25 °C until the color development was sufficient for photometric detection. Thereafter, 25 μL of 1 M H_2_SO_4_ was added to each well, followed by the incubation for 1 min under shaker conditions to stop the reaction. Finally, the absorbance of the samples was measured using an ELISA reader at 450 nm.

### 4.7. cAMP Concentration Assay

The cAMP concentration of D1 cells was measured using the cAMP EIA assay kit (Cayman Chemical Company, Ann Arbor, MI, USA). After the D1 cells were treated with drugs, 1 mM of a cAMP phosphodiesterase inhibitor (IBMX; I-7018; Sigma-Aldrich, St. Louis, MO, USA) was added to the cultured medium for 10 min are 37 °C. The culture plate was washed once with PBS; 0.1 M HCl was added to each well, followed by incubation for 20 min. The cells were scraped off the surface, and the mixture was dissociated to achieve homogeneity. Furthermore, the sample was centrifuged at 1000× *g* for 10 min, and the supernatant was collected in a clean tube. The cAMP concentration assay was performed, as instructed in the manual. Briefly, a 7500-nM bulk standard was diluted to 0.3–750 nM with EIA buffer. A tracer and antibody were added to the standards and samples and incubated at 4 °C overnight. After the incubation, the standards and samples were washed 5 times by PBS, Ellman’s regent was added, and the cells were incubated for 2 h at room temperature. Finally, the absorbance of the samples was measured using an ELISA reader at 405 nm.

### 4.8. Cell Cycle Kinetics Detected Through Flow Cytometry

After 18 h of drug treatment, the cells were detached and flushed with Hank’s buffered solution to prevent aggregation. After centrifugation, the cells were fixed with ice-cold 70% alcohol and incubated at 4 °C for 30 min. Cell membranes were permeated with 0.1% Triton X-100, and RNA was digested with 20 mg/mL of RNase at 37 °C for 1 h. The cells were subsequently stained with 50 mg/mL of propidium iodide (PI, Sigma, St. Louis, MO, USA) in the dark and then filtered (pore size: 41 mm) immediately before analysis. The DNA concentration of an individual cell was measured using a laser flow cytometer. The PI signals were measured on FL3 with a 610/20 nm bandpass filter (EPICS Elite; Beckman Coulter, Hialeah, FL, USA). Aggregation cells and cell debris were excluded. For DNA concentration analysis, live cells were gated with Expo 32™ v1.2 software and used for quantification of cell cycle distribution with WinCycle software (EPICS Elite; Beckman Coulter, Brea, CA, USA).

### 4.9. RNA Extraction and Real-Time Polymerase Chain Reaction

The total RNA from D1 cells was extracted using TRIzol reagent (Cat. 15596026; Invitrogen). First-strand cDNA was synthesized from 1 μg of the total RNA by using a cDNA synthesis kit (Applied Biosystems, Foster City, CA, USA). Quantitative real-time polymerase chain reaction (PCR) was performed in an CFX96 real-time PCR detection system (Bio-Rad Laboratories Inc., Hercules, CA, USA) by using iQSYBR Green Supermix (Bio-Rad Laboratories Inc. Hercules, CA, USA). The reactions were performed in a 25-μL mixture containing cDNA, primers specific for each gene, and iQSYBR Green Supermix. The mRNA levels of cyclins A, B, D1, D2, E1, and E2 and p53, p21, and p27 were determined (Table 1). The following cycling conditions were used: incubation at 94 °C for 1 min, followed by 40 cycles of denaturation at 94 °C for 30 s and annealing and extension at 59 °C for 30 s. The specific PCR products were detected by measuring the fluorescence of SYBR Green. The relative mRNA expression level was normalized to β-actin. The mean gene expression in the control group on day 1 was assigned a value of 1, and the gene expression in each experimental group was calculated relative to this control.

### 4.10. Protein Extraction and Western Blotting

To analyze the protein expression of cell cycle regulators, the protein of D1 cells was extracted from the bone medium at a specific time. The cell protein was extracted using Phosphosafe^™^ extraction reagent (Merck, Darmstadt, Germany). The protein concentration was determined using a protein assay dye reagent (Bio-Rad Laboratories Inc. Hercules, CA, USA). Furthermore, 50 mg of the total protein was separated through 10% sodium dodecyl sulphate polyacrylamide (SDS) gel electrophoresis and transferred to a polyvinylidene fluoride (PVDF) membrane. CREB (N0. 9197; Cell Signaling, Danvers, MA, USA), p-CREB (N0. 9198; Cell Signaling, Danvers, MA, USA), Cyclin D1 (No. 2978; Cell Signaling, Danvers, MA, USA), E1 (No. 4129s; Cell Signaling, Danvers, MA, USA), cyclin-dependent kinase (CDK) 6 (No. 3136; Cell Signaling, Danvers, MA, USA), p-CDK6 (No. sc-293097; Santa Cruz biotechnology, Dallas, TX, USA.), CDK2 (No. ab7954; Abcam, Cambridge, UK), and p-CDK2 (No. 2561S; Cell Signaling, Danvers, MA, USA) were used as primary antibodies. These antibodies were detected using horseradish peroxidase-conjugated anti-mouse, anti-rabbit, or anti-goat secondary antibodies. The secondary antibodies were detected through enhanced chemiluminescence (RPN2235; GE Healthcare) and an AutoChemi image and analysis system (UVP, Upland, CA, USA).

### 4.11. Statistical Analysis

For each study group, the experimental data are presented as the mean ± standard error of mean. Each experiment was performed at least 3 times. Statistical significance was evaluated using 1-way or 2-way analysis of variance (ANOVA), and multiple comparisons were performed using the Scheffe method. The thresholds for significance and high significance were *p* < 0.05 and < 0.01, respectively.

## Figures and Tables

**Table 1:** Primer sequences for real-time PCR. The cycling conditions are as follows: incubation at 94 °C for 1 min, followed by 40 cycles of denaturation at 94 °C for 30 s and annealing and extension at 59 °C for 30 s.

Genes.	Primers
Mouse Cyclin A	F: TGAATCACCACATGCTAT
	R: TAACCTCCATTTCCCTAAG
Mouse Cyclin B	F: TAGGTACTGGAAAAGGTA
	R: GCTTCTCTTCTCTAACAT
Mouse Cyclin D1	F: GCGTACCCTGACACCAATCT
	R: CTCTTCGCACTTCTGCTCCT
Mouse Cyclin D2	F: ACCTGTTGACCATCGAGGAG R: CCAAGAAACGGTCCAGGTAA
Mouse cyclin E1	F: GGAAAATCAGACCACCCAGA R: AGACTTCGCACACCTCCATT
Mouse Cyclin E2	F: TCTCAGGAGACGTTCATCCA R: ACAAAAGGCACCATCCAGTC
Mouse p27	F: CAGAATCATAAGCCCCTGGA R: GGTCCTCAGAGTTTGCCTGA
Mouse Actin	F: CCAACCGTGAAAAGATGACC
	R: ACCAGAGGCATACAGGGACA

## Supplementary Materials

Supplementary materials can be found at https://www.mdpi.com/1422-0067/21/18/6490/s1.
