# Supplementary material for: G Protein-Coupled Estrogen Receptor Mediates Cell Proliferation through the cAMP/PKA/CREB Pathway in Murine Bone Marrow Mesenchymal Stem Cells
Source: Int J Mol Sci. 2020 Sep 5;21(18):6490. doi: 10.3390/ijms21186490 (PMC7555423; doi:10.3390/ijms21186490)
Supplement: Supplementary file 1 [file ijms-21-06490-s001.pdf]

Figure S1

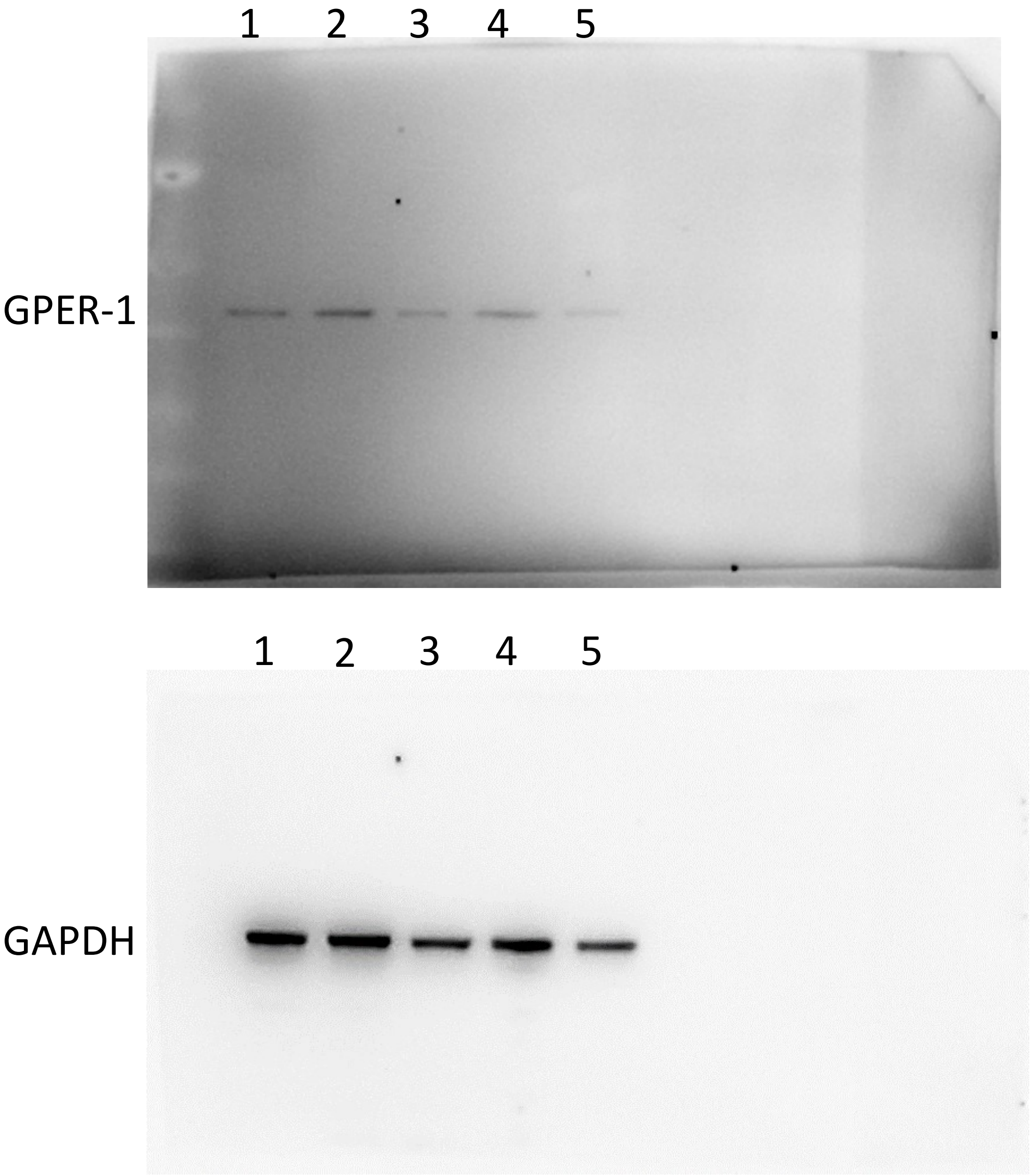

Figure S1. The GPER-1 protein level in BMSCs. Lane 1: 70% cell confluence in bone medium. Lane 2: 100% cell confluence in bone medium. Lane 3-5: D1 cell in osteo-induction medium (OIM) for osteogenic differentiation.
